# Self-medication with antibiotics among out-patient attendants at Madina Polyclinic prior to medical consultation

**DOI:** 10.4314/gmj.v57i4.8

**Published:** 2023-12

**Authors:** Adelaide A Asante, Delia A Bandoh, Ernest Kenu

**Affiliations:** 1 University of Ghana School of Public Health, Department of Epidemiology and Disease Control, College of Health Sciences, Accra, Ghana; 2 Internal Medicine Department, Korle-Bu Teaching Hospital, Accra, Ghana

**Keywords:** self-medication, antibiotics, resistance, OPD attendants, polyclinic

## Abstract

**Objectives:**

The study aimed to determine the prevalence of self-medication with antibiotics among attendants of the Out-patient Department (OPD) at Madina Polyclinic before seeking medical consultation and associated factors.

**Design:**

Cross-sectional study

**Setting:**

The study was conducted at Madina Polyclinic

**Participants:**

The study involved 319 general OPD attendants aged 18 years and above accessing healthcare services at the Madina Polyclinic between May and June 2019.

**Main outcome measures:**

The prevalence of antibiotic self-medication and the factors associated with this practice

**Results:**

From the study, 46.4% (95% CI 40.8%- 52.0%) had self-medicated with antibiotics before presenting for medical consultation at the hospital. Less than half of the respondents (44.5%) had adequate knowledge about the use of antibiotics. Having a tertiary level of education was significantly associated with self-medication (aOR= 8.09, 95% CI 2.31-28.4, p = 0.001), whilst adequate knowledge on the use of antibiotics reduced the odds of self-medication by 53% (aOR= 0.47, 95% CI 0.23- 0.66, p<0.001). The level of education modified the relationship between knowledge and self-medication with antibiotics.

**Conclusion:**

The practice of antibiotic self-medication is rife among OPD attendants. Therefore, adequate public education on the use of antibiotics and the effects of using them inappropriately must be done. The Antimicrobial Resistance (AMR) policy ought to be reinforced and made known to all, especially among the pharmacies that dispense antibiotics indiscriminately.

**Funding:**

None declared

## Introduction

Antibiotics are medicines used for several years to treat bacterial infections. In developing countries, they are essential due to the high rates of infections in these countries. A systematic review and meta-analysis reported that 62% of all antibiotics purchased are bought without prescriptions.^1^ Ocan et al. In 2015, also noted that in developing countries, about 33.4% of persons were involved in self-medication with antibiotics.^2^ likewise, the challenges of limited human resources and healthcare infrastructure in developing countries such as Uganda are fueling the acquisition and use of drugs obtained from pharmacies or medications from leftover drugs, respectively.^3^ A recent study in Ghana showed that nearly a third of hospital attendants had taken antibiotics not prescribed by health personnel before reporting to the hospital.^4^

Donkor et al. in 2012 found that 70% of students at the tertiary level in Accra, Ghana, had self-medicated with antibiotics.^5^

Among developing countries, factors identified to be related to the use of non-prescribed or inappropriate use of antibiotics include paucity of knowledge on the use of antibiotics, low level of education, young age, lack of health facilities, the relatively high cost of consultation at hospitals, and the availability of antibiotics over the counter.^6^–^8^ There has been a gradual increase in the rate at which antibiotics are being used especially in these resource-limited settings and evidence shows that antibiotic resistance is on the rise in these areas where antibiotics are being used irrationally^9^

Antibiotic resistance is the cause of life-threatening infections for which a restricted spectrum of antibiotics can be used in their treatment hence increasing morbidity and mortality, prolonging admission in the hospital and increasing the cost of health care.^7^

Adverse drug reactions may occur from the irrational use of drugs including antibiotics as pharmacies may fail to ask about allergies or the simultaneous use of other medications which may cause undesirable drug interactions.^10^ The irrational use of antibiotics may alter the clinical presentation and natural history of diseases, leading to diagnostic delays.^10^

Self-medication has been identified as one of the contributors to antimicrobial resistance in low- and middle-income countries.^11^ Thus, tackling self-medication with antibiotics will help minimize the progression of antimicrobial resistance. Even though the prevalence of self-medication with antibiotics is known among tertiary students in greater Accra, inhabitants in the Cape Coast metropolis and Kumasi^4^,^5^,^12^, little is known about the prevalence of antibiotics use in Greater Accra, the capital city. Knowledge of the prevailing pattern and understanding of the patient-related factors that underlie the practice of self-medication is needed. We assessed self-medication with antibiotics before medical consultation in Madina Polyclinic, a health facility surrounded by several tertiary institutions including the largest tertiary institution in the country and several other tertiary institutions.

## Methods

### Study design

A facility-based cross-sectional study was conducted as patients accessed health care in Madina Polyclinic, Greater Accra from May to June 2019. Quantitative data was collected through self-filled questionnaires.

### Study area

The study was conducted at the Madina Polyclinic Rawlings Circle located in the La- Nkwantanang municipal district, one of the sixteen districts in the Greater Accra region of Ghana. The municipality has thirty-nine health facilities, two of which are government polyclinics, namely Madina polyclinic-Kekele and Madina Polyclinic Rawlings Circle. The study was conducted in the latter. Services provided at the facility include General OPD, laboratory, ultrasound, DOTS, ART clinic, Antenatal, reproductive and child health clinics, dental, Ear, Nose and Throat (ENT) and eye care. An average of about 100-120 patients are seen daily, for various conditions.

### Study population

All general OPD attendants aged 18 years and above who accessed health care services at Madina Polyclinic Rawlings Circle between May and June 2019 formed the study population.

### Sample size determination

The sample size for the study was calculated based on the prevalence of self-medication in a previous study done in a clinic Kumasi, Ghana where the prevalence of self-medication with antibiotics was 75%.^13^ The sample size for this study was calculated as follows:

n = Z2 × PQ/d2, where n represents the desired sample size, Z is the normal standard deviation, whose value at 95.0 % confidence level is 1.96, P = prevalence of antibiotic self-medication in the above-mentioned study was 0.75, Q = 1-P =0.25, and d = the set margin of error: 0.05. The minimum sample size obtained was 289. This number was adjusted upwards by 10 % to allow likely non-response or recording errors. The final sample size was 318.

### Sampling technique

A simple random sampling technique was used. Folded pieces of paper with ‘yes’ and ‘no’ responses were put in a container, from which the patients were asked to pick. The patients who chose ‘yes’ and satisfied the inclusion criteria, were recruited to participate in the study. On average, 20 respondents were obtained each day during the study period. Patients whose vitals had been checked and arranged in the order in which they would see the doctor were involved in this selection process for the study. The procedure was repeated each day until the sample size of 319 was achieved.

### Data collection

Questionnaires were distributed to participants to fill out after obtaining approved written informed consent. This was done at the OPD and respondents were interviewed before seeing the doctor. Participants who were unable to fill out the responses by themselves were assisted by the research assistants. The questions were in the English language and translation was done by research assistants to a language best understood by the respondents. Patients who consented were taken outside the general OPD to a place the interview could not be overheard.

The questionnaire was designed by adapting parts of a standardized questionnaire used by WHO in assessing self-medication with antibiotics in a Multi-Country Public Awareness Survey.^14^ The questionnaire was well structured and made up of close-ended questions. There were three parts to the questionnaire. These were demographic characteristics, information from those who had self-medicated with antibiotics, reasons for self-medicating with antibiotics, symptoms, types of antibiotics used and, sources of antibiotics and knowledge questions. The level of knowledge about antibiotics was assessed using nine close-ended questions. Respondents who answered five or more questions correctly were categorized as having adequate knowledge.

Pre-testing was done at Taifa Polyclinic for reliability and efficiency. Corrections were made based on the results of the pre-test. The modified questionnaire was then used for the data collection at the study site. Three research assistants were trained in administering questionnaires in both Twi and English. Translation and backtranslation were done during training. This ensured that all questions were asked in the same way. Each day, questionnaires were checked to ensure answers were correctly provided. The assistants addressed issues where gaps were identified by referring to field notes taken during interviews.

### Data processing and analysis

The data was entered in a Microsoft Excel Spreadsheet for Windows 10. Data were cleaned, checked, coded and imported into STATA software version 15 for statistical analysis. Continuous variables were summarized as mean ± standard deviation, and categorical variables were summarized as frequencies. The results of the frequency distributions from the collected data were presented using tables and graphs.

Simple logistic regression and multivariate analyses were done to determine the relationship between the independent variables and antibiotic self-medication. Firstly, for each independent variable (age, sex, occupational status, educational status, and level of knowledge), a simple logistic regression analysis was performed to give the p-value for their coefficient, which explained the statistical significance of the selected independent variables. The odds ratios (OR) were calculated, and statistical significance was accepted at p≤0.05. Finally, regardless of their significance, a multivariate analysis was carried out for the same independent variables used in the simple logistic regression. The adjusted odds ratios (aOR) were again determined, and significance was accepted at p≤0.05.

### Ethical consideration

Ethical clearance was obtained from the Ghana Health Service Ethics Review Board (GHS-ERC 024/01/19). Formal permission was obtained from the La-Nkwantanang Municipal Health Directorate and the administrator of the polyclinic. Informed consent was obtained from the patients recruited into the study before distributing the questionnaires. Participants were assigned unique codes to ensure anonymity. Data was kept on password-protected devices and made available only to the research team members on issues relating to the work.

## Results

### Demographic characteristics of respondents

The respondents were mostly females, 60.2% (192/319). The mean age of the respondents was 35.6 ± 13.6 years. Table 1 shows the demographic characteristics of the respondents.

**Table 1:** Socio-demographic characteristics of respondents

Variable	Frequency (%) (n=319)
**Sex**	
**Female**	192 (60.2)
**Male**	127 (39.8)
**Occupational status**	
**Sales And Service**	78 (24.5)
**Agriculture**	6 (1.9)
**Skilled Manual Labour**	22 (6.9)
**Professional/Technical/Managerial**	83 (26.0)
**Unskilled Manual Labour**	27 (8.5)
**Clerk**	4(1.2)
**Student**	60 (18.8)
**Not Employed**	39 (12.2)
**Educational Status**	
**No Education**	17 (5.3)
**Primary**	25 (7.8)
**Junior High School**	61 (19.1)
**Senior High School**	100 (31.4)
**Tertiary/University**	115 (36.4)
**Marital Status**	
**Single**	170 (53.3)
**Married**	123 (39.6)
**Divorced**	10 (3.1)
**Widowed**	16 (5.0)
**Mean Age (years)**	35.6 ± 13.6

### Prevalence of self-medication with antibiotics

Of the 319 people interviewed, 148 (46.4%, 95% CI 40.8% to 52.0%) reported self-medicating with antibiotics before hospital consultation. Amoxicillin was used most frequently, 66 (56.9%), followed by ciprofloxacin 17 (14.7%) and ampicillin 12 (10.4%).

### Reasons for self-medicating with antibiotics

Previous successful use of antibiotics made up more than three-fourths (93/114) of the response for self-medicating with antibiotics. More than half (78/148) reported spending long at the hospital. Distance to the health facility was the least given reason for self-medicating (Table 2).

**Table 2:** Reasons for self-medicating with antibiotics

Reason	Frequency (%)
**Previous successful use**	93 (81.6)
**Spending long hours at a health facility**	78 (52.7)
**Less than 1 hour**	2 (2.6)
**1-2 hours**	15 (19.2)
**2-3 hours**	27 (34.6)
**More than 3 hours**	34 (43.6)
**Relative/friend's recommendation**	44 (38.9)
**High cost of hospital bills**	43 (38.6)
**Busy schedule**	36 (30.6)
**Distance to hospital**	19 (17.6)

*Multiple choices responses

### Sources of antibiotics in self-medicating

Amongst the 148 patients who had self-medicated before reporting to the hospital, most of the respondents 79.3%, obtained the drugs from the pharmacy. Only 4.7% of them obtained them as leftovers from previous use.

### Knowledge about antibiotics

Almost 60% of the respondents reported that antibiotics are used to treat bacterial infections; 19.1% reported that antibiotics cannot be used for viral infections and 31% knew that antibiotics cannot be used to treat all infections. Majority (72%) deemed it right to share antibiotics with relatives and 16% reported that antibiotics could be used for recurring symptoms (Table 3).

**Table 3:** Respondents' knowledge on antibiotic use

Variable	Frequency(%)
**Antibiotics are used to treat bacterial infections**	
**Yes**	187 (58.6)
**No**	14 (4.4)
**Don't know**	118 (37.0)
**Antibiotics are used to treat viral infections**	
**Yes**	100 (31)
**No**	61 (19.1)
**Don't know**	158 (49.5)
**Antibiotics are used to treat all infections**	
**Yes**	79 (24.8)
**No**	99 (31.0)
**Don't know**	141 (44.2)
**You stop antibiotics when you feel better during the course**	
**Yes**	152 (47.7)
**No**	124 (38.9)
**Don't know**	43 (13.5)
**You stop taking antibiotics after you have completed a course as directed**	
**Yes**	211 (66.1)
**No**	69 (21.6)
**Don't know**	39 (12.2)
**You can share antibiotics with relatives**	
**Yes**	51 (72.1)
**No**	230 (16.0)
**Don't know**	38 (11.9)
**You can self-medicate with previously used antibiotics**	
**Yes**	110 (34.5)
**No**	166 (52.0)
**Don't know**	43 (13.5)
**Side effects of antibiotics**	
**No side effects**	112 (35.1)
**Has side effect**	128 (40.1)
**Don't know**	79 (24.8)
**Overuse leads to resistance**	
**Yes**	157 (49.2)
**No**	34 (10.7)
**Don't know**	1 28 (40.1)

### Level of knowledge about antibiotics

Forty-four percent (142/319) of the respondents had adequate knowledge about antibiotics whilst 55.5% (177/319) had inadequate knowledge.

### Factors associated with self-medication using antibiotics

Having a tertiary education (cOR=3.23, 95%1.07-9.78, p<0.001) and adequate level of knowledge (cOR=0.63, 95% CI 0.41-0.99, p-value<0.05) were found to be significantly associated to self-medication.

### Symptoms treated with antibiotics

**Figure 1:**
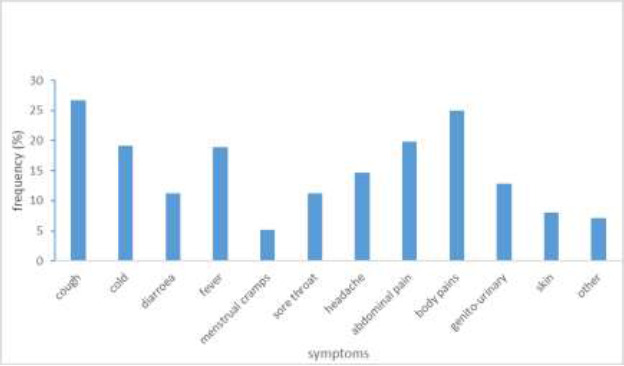
Conditions respondents reported self-medicating with antibiotics

The commonest symptom for which antibiotics were used in self-medicating was a cough, representing 26.7% of the responses. Body pain was the next symptom (Figure1).

Controlling for age, sex, occupational status, educational status and marital status, the odds of self-medication amongst respondents with adequate knowledge about antibiotics was reduced by 53% compared to those who had inadequate knowledge and this is statistically significant (aOR= 0.47, 95% CI 0.23- 0.66, p<0.001) (Table 4).

**Table 4:** Binary and multivariable logistic regression analysis showing factors associated with self-medication with antibiotics

Variable	Self-medication	Non-self- medication	cOR (95%CI)	aOR (95%CI)
**Educational Status**				
**No Education**	5(29.4)	12(70.6)	1	1
**Primary**	10(40.0)	25(60.0)	1.60(0.43-5.96)	1.77(0.45- 6.96)
**Junior High School**	25(41.0)	36(59.0)	1.67(0.52-5.32)	1.89(0.56-6.40)
**Senior High School**	41(41.0)	59(59.0)	1.67(0.55-5.09)	2.87(0.87-9.50)
**Tertiary/University**	66(57.4)	49(42.6)	3.23(1.07-9.78)*	8.09(2.31-28.41)**
**Marital Status**				
**Single**	80(47.1)	90(52.9)	1	1
**Married**	56(45.5)	67(54.5)	0.94(0.59-1.50)	1.33(0.71-2.49)
**Divorced**	6(60.0)	4(40.0)	1.69(0.46-6.19)	2.53(0.59-10.87)
**Widowed**	6(37.5)	10(62.5)	0.68(0.23-1.94)	1.05(0.27-4.00)
**Level of Knowledge**				
**Inadequate**	91(51.4)	86(48.6)	1	1
**Adequate**	57(40.1)	85(59.9)	0.63(0.41-0.99)*	0.47(0.23-0.66)**

* p<0.05

** p<0.001

The odds of self-medication was eight times greater among respondents who had attained tertiary level of education compared to those who had no education and this association was statistically significant (aOR= 8.09, 95% CI 2.31-28.4, p = 0.001).

From Table 5, stratifying by educational level, among those with a lower level of education, the odds of self-medication was significantly reduced by 70% among those who had adequate knowledge compared to those who did not whereas among those with a higher level of education, this was reduced by only 32% although this did not attain statistical significance.

**Table 5:** Relationship between adequate knowledge of the use of antibiotics and self-medication with antibiotics, stratifying by educational status

Knowledge measure	High level of education		Odds ratio (95% CI)	Low level of education		Odds ratio (95% CI)
	Self-medication	No self-medication		Self-medication	No self-medication	
**Adequate knowledge**	51(47.6)	62(57.4)	0.68(0.38-1.19)	6(15.0)	23(36.5)	0.30(0.09-0.90)*
**Inadequate knowledge**	56(52.3)	46(42.6)		34(85.0)	40(63.5)	

## Discussion

From the study, the proportion of patients who had self-medicated with antibiotics at Madina polyclinic was 46.4% similar to findings from hospital-based studies done in Kenya and Cameroun where 48% and 41.9% of patients at the OPD had taken antibiotics without prescription, respectively.^15^,^16^ Earlier studies done in Ghana, almost a decade ago showed a relatively higher prevalence of antibiotic self-medication (71%) in the Cape Coast metropolis of Ghana and a 70% prevalence among tertiary students in Accra.^5^,^12^ The reduction in the prevalence in Ghana recently could be attributed to the introduction of the antimicrobial policy in 2018 which has probably reduced the ease with which patients acquire nonprescribed antibiotics as some pharmacies have also become more vigilant in dispensing these nonprescribed medications.

Less than half (44.5%) of the respondents had adequate knowledge of the uses of antibiotics. A similar study in South Africa identified 53% of the respondents as having adequate knowledge. 17 The knowledge base of most people on what antibiotics are used for is quite low, which contributes to their irrational use.

The study has exposed deficits in the respondents' knowledge about antibiotics. A common perception exists even amongst the elite and well-educated that antibiotics are very potent medications that can cure all infections.

Here, majority of the respondents were unaware that antibiotics could not be used against all infections. Almost half knew that the management of infections caused by bacteria is using antibiotics. Similar to findings in Cameroon, over 87% of respondents agreed that antibiotics can treat all infections and 43.7% knew antibiotics could be used against bacteria.^18^ These results could also be because fewer people in the general population know the differences between these microorganisms. Oh et al. noted that when providing medical advice to laypersons on the infectious causes of disease, the term ‘germs’ is often erroneously used instead of terms like viruses or bacteria hence the lack of knowledge that different antimicrobials are used for different infectious diseases from different causes.^19^

Less than half of the patients deemed it right to stop taking antibiotics once symptoms improved on antibiotic treatment despite advice on treatment duration given by a prescriber. This practice shows a lack of understanding in adhering to antibiotic treatment regimens. In a survey by the World Health Organization, as high as 62% of respondents stopped taking their drugs upon feeling better. 14 Comparably, in Kenya, 84% had stopped with improved symptoms.^19^,^20^ Failure to complete an antibiotic course contributes to antibiotic resistance.^14^ The best way to avoid resistance is therefore to adhere to the treatment course.

Our findings showed that more than a third knew about the side effects of antibiotics whilst 40.1% reported no side effects. Likewise, only 20% of participants in a study in South Africa were aware of the side effects of self-medication with antibiotics whereas as high as 61% disagreed.^17^ On the other hand, in a similar research done in Tanzania, up to 98% knew that the inappropriate use of antibiotics could be associated with harmful side effects.^21^ The poor knowledge about the side effects of antibiotics could be due to the weak reporting system that we have in the country regarding drug reactions hence there is little documented data about these drug reactions to sensitize the general public. This finding from the study is problematic as many people have been admitted and treated for life-threatening drug reactions after taking seemingly “common” antibiotics. 22

The study discovered that a higher level of education was a significant predictor of antibiotic self-education. Those who had completed tertiary/ university school were likely to self-medicate and this was statistically significant on both the bivariate and multivariate analyses. This is consistent with findings in Cameroon where university graduates had double the odds of self-medicating with antibiotics compared to those with lower educational levels.^18^ Studies in Jordan also revealed a similar pattern where higher education was associated with antibiotic self-medication.^23^ Similarly in Europe, self-medication with antibiotics was associated significantly with individuals who were well educated.^24^ This could be because this group of people may have some form of understanding of diseases and medications hence a decreased tendency to seek physician or prescriber's advice on the treatment of certain ailments. Again, with the improvement in technology and increased access to information through the internet, these educated people may resort to the internet which might not necessarily provide the correct information. In contrast, illiterates in Ethiopia were four times more likely to self-medicate compared to those who had completed university. 6 Nepal et al. found that individuals who had attained higher education in South East Asia were unlikely to self-medicate with antibiotics.^25^

The study revealed that having adequate knowledge about the use of antibiotics reduced the odds of self-medication in both bivariate and multivariate analyses respectively. According to Ocan et al., the odds of self-medication with antibiotics by patients who knew about antibiotics was reduced by 30%.^3^ In South Africa, respondents with adequate knowledge about antibiotics were less likely to self-medicate. 17 People who have adequate knowledge consider it a safer option to discuss antibiotics with a health provider before starting treatment. Contradictory findings were reported in the United Kingdom where adequate knowledge about antibiotics was associated with a two times increased likelihood of self-medication.^26^ This shows that although knowledge is important, there could be other factors that contribute to the decision to self-medicate with antibiotics and this must be researched into. From the results, the level of education is an effect modifier of the relationship between the level of knowledge on the use of antibiotics and self-medication.

From the study, the commonest reason for self-medicating with antibiotics was previous successful use. This is consistent with studies done in Ghana, Jordan and Kuwait where most people self-medicated with antibiotics that had been prescribed for an ailment they had suffered previously.^2^,^23^,^27^ Most persons rely on the fact that because they have effectively treated certain illnesses with antibiotics, subsequent diseases can be treated with the same antibiotics without a physician's consult. This is an erroneous thought as most of them lack the understanding of the pathophysiology of disease processes and are only exposing themselves to the adverse consequences of the medication instead.

About half of the patients admitted that spending long hours at the hospital influenced them to self-medicate with antibiotics.

For instance, 85% of university students in China stated that it was more convenient to self-medicate than spend almost the whole day at the hospital. 28 Likewise, long waiting time at health facilities was a significant predictor of self-medication to antibiotics in Northern Uganda.^3^

The limitation of this study is that it was carried out in a single facility and may have a restricted representation to the rest of the country. Although a list was provided to help the respondents identify the antibiotics taken, the type of antibiotic may not have been accurately recalled. This could impact the nature of the results obtained.

## Conclusion

From this study, a significant proportion of the OPD attendants (46.4%) had self-medicated with antibiotics before reporting to the hospital. Knowledge about the use of antibiotics was inadequate. In addition, the level of education about the use of antibiotics was low as only 44.5% had adequate knowledge about their use. There should be public education on antimicrobial resistance as self-medication with antibiotics contributes to this menace. Antibiotic resistance is a looming pandemic and if not managed well could have serious consequences worldwide.

## References

[1] Auta A, Hadi MA, Oga E (2019). Global access to antibiotics without prescription in community pharmacies: A systematic review and meta-analysis. J Infect.

[2] Ocan Moses, Ekwaro A (2015). Obuku, Freddie Bwanga, Dickens Akena4, Sennono Richard JO-O and CO. Household antimicrobial self-medication: a systematic review and meta-analysis of the burden, risk factors and outcomes in developing countries. Kardiol Pol.

[3] Ocan M, Bwanga F, Bbosa GS (2014). Patterns and Predictors of Self-Medication in Northern Uganda. PLoS One.

[4] Donkor GY, Dontoh E, Owusu-Ofori A (2019). A cross-sectional study on the prevalence of antibiotic use prior to laboratory tests at two Ghanaian hospitals. PLoS One.

[5] Donkor ES, Tetteh-Quarcoo PB, Nartey P, Agyeman IO (2012). Self-medication practices with antibiotics among tertiary level students in Accra, Ghana: A cross-sectional study. Int J Environ Res Public Health.

[6] Gebeyehu E, Bantie L, Azage M (2015). Inappropriate use of antibiotics and its associated factors among urban and rural communities of Bahir Dar city administration, northwest Ethiopia. PLoS One.

[7] Kariuki S, Dougan G (2014). Antibacterial resistance in sub-Saharan Africa: An underestimated emergency. Ann N Y Acad Sci.

[8] Surji KM (2016). Antibiotics Misuse and Factors Leading to Its' Abuse in Kurdistan Region. J Heal Med Nurs.

[9] Morgan DJ, Okeke IN, Laxminarayan R, Perencevich EN (2013). Non prescription use of antibiotics a worldwide systematic review. Nih.

[10] Akinyandenu O, Akinyandenu A (2014). Irrational use and non-prescription sale of antibiotics in Nigeria: A need for change. J Sci Innov Res JSIR.

[11] de Lima Procopio RE, da Silva IR, Martins MK, de Azevedo JL, de Araujo JM (2012). Antibiotics produced by Streptomyces. Brazilian J Infect Dis.

[12] Tagoe DNA, Attah CO (2012). A Study of Antibiotic Use and Abuse in Ghana: a case study of the Cape Coast Metropolis. Internet J Heal.

[13] Adu-Sarkodie YA (1997). Antimicrobial self medication in patients attending a sexually transmitted diseases clinic. Int J STD AIDS.

[14] World Health Organization (WHO) (2015). Antibiotic Resistance: Multi-Country Public Awareness Survey. Antibiot Resist MULTI-COUNTRY PUBLIC Aware Surv.

[15] Ngigi CK (2016). Self medication with antibiotics prior to seeking treatment among adult patients attending out-patient department at Gatundu Sub-County Hospital, Kiambu County, Kenya.

[16] Ngu RC, Feteh VF, Kika BT, F EKN, Ayeah CM, Chifor T, Njim T, Fankem AM, Yengo FKF (2018). Prevalence and Determinants of Antibiotic Self-Medication among Adult Patients with Respiratory Tract Infections in the Mboppi Baptist Hospital, Douala, Cameroon: A Cross-Sectional Study. Diseases.

[17] Ramchurren K, Balakrishna Y, Mahomed S (2018). Patients' knowledge, attitudes and practices regarding antibiotic use at a regional hospital in KwaZulu-Natal, South Africa 2017. South African J Infect Dis.

[18] Ekambi GAE, Ebongue CO, Penda C, Nga EN, Mpondo EM, Moukokoid CEE (2019). Knowledge, practices and attitudes on antibiotics use in Cameroon: Self-medication and prescription survey among children, adolescents and adults in private pharmacies. PLoS One.

[19] Oh AL, Hassali MA, Al-haddad MS, Azhar S, Sulaiman S (2010). Public knowledge and attitudes towards antibiotic usage: a cross-sectional study among the general public in the state of Penang, Malaysia. J Infect Dev Countries.

[20] Omulo S, Thumbi SM, Lockwood S (2017). Evidence of superficial knowledge regarding antibiotics and their use: Results of two cross-sectional surveys in an urban informal settlement in Kenya.

[21] Kumburu HH, Sonda TB, Mwanziva CE (2018). Prevalence, determinants and knowledge of antibacterial self-medication : A cross sectional study in North-eastern Tanzania. PLoS One.

[22] Shehab N, Patel PR, Srinivasan A, Budnitz DS (2008). Emergency Department Visits for Antibiotic-Associated Adverse Events. Clin Infect Dis.

[23] Al-Azzam SI, Al-Husein BA, Alzoubi F, Masadeh MM, Al-Horani MAS (2007). Self-medication with antibiotics in Jordanian population. Int J Occup Med Environ Health.

[24] Grigoryan L, Burgerhof JGM, Haaijer-ruskamp FM (2007). Is self-medication with antibiotics in Europe driven by prescribed use ?. J Antimicrob Chemother.

[25] Nepal G, Bhatta S (2018). Self-medication with Antibiotics in WHO Southeast Asian Region: A Systematic Review. Cureus.

[26] Mcnulty CAM, Boyle P, Nichols T, Clappison P, Davey P, Dd D (2007). Don't wear me out — the public's knowledge of and attitudes to antibiotic use. J Antimicrob Chemother.

[27] Awad AI, Aboud EA (2015). Knowledge, Attitude and Practice towards Antibiotic Use among the Public in Kuwait. PLoS One.

[28] Zhu X, Pan H, Yang Z, Cui B, Zhang D, Ba-thein W (2015). Self-medication practices with antibiotics among Chinese university students. Public Health.

